# Atrial arrhythmia prevalence and characteristics for human immunodeficiency virus-infected persons and matched uninfected controls

**DOI:** 10.1371/journal.pone.0194754

**Published:** 2018-03-20

**Authors:** Jes M. Sanders, Alexandra B. Steverson, Anna E. Pawlowski, Daniel Schneider, Chad J. Achenbach, Donald M. Lloyd-Jones, Matthew J. Feinstein

**Affiliations:** 1 Northwestern University Feinberg School of Medicine, Chicago, IL, United States of America; 2 Northwestern Medicine Enterprise Data Warehouse, Northwestern University, Chicago, IL, United States of America; 3 Division of Infectious Diseases, Department of Medicine, Northwestern University Feinberg School of Medicine, Chicago, IL, United States of America; 4 Division of Cardiovascular Disease, Department of Medicine, Northwestern University Feinberg School of Medicine, Chicago, IL, United States of America; 5 Department of Preventive Medicine, Northwestern University Feinberg School of Medicine, Chicago, IL, United States of America; Azienda Ospedaliera Universitaria di Perugia, ITALY

## Abstract

**Background:**

Human Immunodeficiency Virus-Infected (HIV+) persons have elevated risks for various manifestations of cardiovascular disease (CVD). No studies to our knowledge have compared atrial fibrillation (AF) and atrial flutter (AFL) prevalence and associated characteristics for HIV+ persons and matched uninfected controls.

**Methods and findings:**

Persons with diagnoses of HIV receiving care at a large urban academic medical center were frequency-matched 1:2 on age, sex, race, zip code, and clinic location with uninfected persons. Possible AF/AFL was screened for using administrative codes and diagnoses of AF/AFL were subsequently adjudicated using electrocardiography and physician notes; adjudication was performed given the inconsistent validity of administrative code-derived AF diagnoses found in previous studies. There were 101 confirmed AF/AFL cases (2.00%) among 5,052 HIV+ patients and 159 confirmed AF/AFL cases (1.57%) among 10,121 uninfected controls [Odds Ratio (OR) 1.27, 95% Confidence Interval (CI) 0.99–1.64; p = 0.056]. The association between HIV serostatus and AF/AFL was attenuated after adjustment for demographics and CVD risk factors. Among HIV+ persons, nadir CD4+ T cell count <200 cells/mm^3^ was associated with approximately twofold elevated odds of AF/AFL even after adjustment for demographics and CVD risk factors (Multivariable-adjusted OR 1.98, 95% CI 1.21–3.25). There was no significant association between log_10_ of peak HIV viral load and AF/AFL (Multivariable-adjusted OR 1.03, 95% CI 0.86–1.24). Older age, diabetes, hypertension, and chronic obstructive pulmonary disease were associated with similarly elevated odds of AF/AFL for HIV+ persons and uninfected controls.

**Conclusion:**

HIV-related immunosuppression (nadir CD4 T cell count <200 cells/mm^3^) and traditional CVD risk factors are associated with significantly elevated odds of AF/AFL among HIV+ persons. Although atrial fibrillation and flutter was more common among HIV+ versus uninfected persons in this cohort, this difference was attenuated by adjustment for demographics and CVD risk factors.

## Introduction

The longevity of human immunodeficiency virus-infected (HIV+) persons has increased due to effective and available antiretroviral therapy (ART) [1–8]. As the HIV+ population ages, HIV+ persons are experiencing an increasing burden of comorbidities, including cardiovascular diseases (CVDs) [9–11]. Myocardial infarction, arrhythmias, heart failure, and sudden cardiac death all appear to occur more frequently for HIV+ versus uninfected persons [10, 12–15]. However, although epidemiologic data suggest elevated risks for arrhythmias and sudden cardiac death among HIV+ persons, clinical characteristics and mechanisms associated with these risks are not well understood. A previous analysis of HIV+ persons in the Veterans Affairs (VA) HIV Clinical Case Registry (which is >97% male) used International Classification of Disease-9 (ICD-9) codes to identify likely AF/AFL diagnoses and found that high HIV viral load and low CD4+ T cell count (CD4 count) were associated with significantly elevated incidence of AF/AFL [14]. However, no previous studies to our knowledge have compared AF/AFL for HIV+ persons and uninfected controls. Similarly, although administrative codes may have a positive predictive value as low as 70% for identifying AF, no previous studies to our knowledge have adjudicated AF/AFL diagnoses among HIV+ persons [16].

In this study, we compared the prevalence of physician-adjudicated AF/AFL among HIV+ persons and matched uninfected controls in a large electronic cohort and evaluated factors associated with AF/AFL for HIV+ persons. We hypothesized that: 1) AF/AFL is more common among HIV+ persons compared with uninfected controls and 2) Traditional CVD risk factors, lower nadir CD4 count, and higher peak HIV viral load are associated with greater risk for AF/AFL among HIV+ persons.

## Methods

### Study population

We used a large electronic health record (EHR)-based cohort of HIV-infected persons and matched uninfected controls receiving care at a large urban academic center: the HIV Electronic Comprehensive Cohort of CVD Complications (HIVE-4CVD). During the period of observation from January 1, 2000 to July 12, 2016, we identified HIV+ adults aged 18 years and older using the following validated definition[17]: 1) positive HIV-1 antibody or serology, 2) positive (>0) HIV viral load, or 3) at least three orders of HIV viral load and a CD4 T cell count ordered on the same date. Uninfected controls were frequency-matched with HIV+ persons using a propensity score incorporating age, sex, race, zip-code, and clinic location. The HIVE-4CVD cohort creation and this protocol were approved by the Institutional Review Board at Northwestern University (Chicago, IL). A waiver of consent was applied by the Institutional Review Board due to the infeasibility of contacting patients in this retrospective analysis of already-collected data and chart review.

### Demographics and clinical covariates

Data from the first clinical encounter for each patient were used to determine baseline age, sex, and race. Body-mass index (BMI) was defined as weight (kg) divided by the square of height (m^2^) at the first concurrent height and weight measurement for each patient recorded in the NMEDW. Smoking data were not available for HIV+ persons and inconsistently available for uninfected persons in our cohort and were therefore no able to be included in analyses. Hypertension was defined by ICD-9 (401–405) or ICD-10 (I10-I15) codes, and due to heterogeneity of inpatient and outpatient visits in this cohort with the potential for systematic differences in measurement, observed blood pressure values were not used in the definition of hypertension [12]. Diagnoses of diabetes were described as possible in an individual with ICD-9 or ICD-10 code (ICD-9-CM 249–250, ICD-10-CM E08-11, ICD-10-CM E13), and positive if an individual had an ICD-9 or ICD-10 code *and* (1) a hemoglobin A1C >6.4% or (2) was prescribed any diabetic medication [18]. Chronic obstructive pulmonary disease (COPD) diagnoses were identified using ICD9 codes (ICD-9-CM 491–492, 496) or ICD-10-CM codes (ICD-10-CM J43-J44). CHADS score was tabulated for both HIV+ and HIV- patients using these diagnosis codes as well as stroke diagnosis codes (ICD-9 (433–436) or ICD-10 (I60-I63)) [19, 20]. Hepatitis C virus infection was defined as a positive result for a hepatitis C virus antibody test or detectable viremia [21]. CD4 lymphocyte counts, HIV-1 RNA levels, and use of ART were also collected for HIV+ patients. We evaluated nadir CD4 count and peak HIV viral load, rather than baseline, most recent, or as a time-updated covariate, due to heterogeneity in the timing and setting of CD4 count and HIV viral load measurement in our EHR-based study.

### Atrial fibrillation/atrial flutter screen and adjudication

Physician-adjudicated atrial fibrillation/atrial flutter was the primary outcome of interest in this study. In the initial screen, patients were considered to have possible AF/AFL based on the following criteria: 1) mention of AF/AFL in a physician note or problem list from inpatient or outpatient encounters, using “atrial fibrillation” and “atrial flutter” as search terms; 2) any ICD-9-CM or ICD-10-CM diagnosis code from inpatient or outpatient encounters consistent with atrial fibrillation (427.31, I48.0, I48.1, I48.2, I48.91) or atrial flutter (427.32; I18.3, I48.4, I48.92); and/or 3) any encounter with CPT procedural codes consistent with AF/AFL-related procedures (93653, 93655, 93656, 93657). Two independent investigators then performed chart review for each person with possible AF/AFL and identified persons with confirmed AF/AFL based on electrocardiographic evidence of AF/AFL and/or documentation in physician notes confirming AF/AFL.

### Statistical analyses

We first compared demographics and clinical covariates of HIV+ persons and uninfected controls in our cohort. Next, we compared the prevalence of adjudicated AF/AFL for HIV+ persons and uninfected controls patients diagnosed with AF/AFL using multivariable-adjusted logistic regression. We then evaluated associations of HIV-specific exposures (nadir CD4 count <200 cells/mm3 and log-transformed peak HIV viral load) with AF/AFL for HIV+ persons using multivariable-adjusted logistic regression. Multivariate models were performed unadjusted (Model 1); adjusted for age, sex, and race (Model 2; persons of black, white, or Hispanic race were included due to incomplete data for other races in this cohort); and additionally adjusted for BMI, hypertension, diabetes, and COPD (Model 3). For a sensitivity analysis, we added duration of antiretroviral therapy (ART) as a covariate to Model 3; ART duration for each HIV+ person was approximated as the time between the earliest noted date of ART prescription and either most recent follow up or death.

## Results

Demographics and clinical covariates of the HIVE-4CVD cohort are shown in Table 1. As expected in a frequency-matched cohort in which age and demographics were criteria for matching, the mean age in both HIV+ and HIV- groups was 48.2, and a similar proportion of patients in each group was male (82.5% and 82.8%, respectively). Patients diagnosed with HIV were more likely to have a lower BMI, high total cholesterol and low HDL cholesterol, hypertension, diabetes, CHD, MI, COPD, and stroke (p<0.01). Of the 5,052 HIV+ persons, 133 were identified as having possible AF/AFL from the primary screen, of which 101 (2.00%) were confirmed to have AF/AFL by physician adjudication. Among uninfected controls, 208 out of 10,121 persons were identified as having possible AF/AFL, of which 159 (1.57%) were confirmed to have AF/AFL by physician adjudication. This borderline-significant difference in AF/AFL prevalence for HIV+ versus uninfected persons (OR 1.28, 95% CI 0.996–1.65; p = 0.056) was attenuated after adjustment for demographics and CVD risk factors (Table 2).

**Table 1:** Demographic and clinical characteristics of HIV+ persons and frequency-matched controls.

		HIV+ (N = 5052)	Controls (N = 10121)	P-value
Age (years)		48.2 ± 11.6	48.2 ± 11.4	0.68
	18–30, N (%)	331 (6.6%)	647 (6.4%)	0.98
	31–40	921 (18.3%)	1871 (18.5%)	
	41–50	1451 (28.8%)	2886 (28.5%)	
	51–60	1615 (32.0%)	3291 (32.5%)	
	61–70	590 (11.7%)	1159 (11.4%)	
	>70	133 (2.6%)	267 (2.6%)	
Race (N, %)				<0.01
	White	1680 (34.5%)	3207 (39.9%)	
	Black	1573 (32.3%)	2698 (33.5%)	
	Hispanic	173 (3.6%)	359 (4.5%)	
	Other/Unknown	1438 (29.6%)	1783 (22.2%)	
Male (N, %)		4167 (82.5%)	8381 (82.8%)	0.13
Body-Mass Index (kg/m2)		25.9 ± 5.6	28.6 ± 6.5	<0.01
Total cholesterol (mg/dl)		202.2 ± 59.1	194.5 ± 50.6	<0.01
HDL cholesterol (mg/dl)		34.3 ± 13.1	42.9 ± 14.1	<0.01
Hepatitis C Virus (N, %)		425 (8.4%)	90 (0.9%)	<0.01
Hypertension (N, %)		1674 (33.1%)	1893 (18.7%)	<0.01
Diabetes (N, %)		463 (9.2%)	603 (6.0%)	<0.01
Coronary Heart Disease (N, %)		728 (14.4%)	704 (7.0%)	<0.01
Myocardial Infarction (N, %)		571 (11.3%)	502 (5.0%)	<0.01
Chronic Obstructive Pulmonary Disease (N, %)		305 (6.0%)	223 (2.2%)	<0.01
Stroke, N (%)		316 (6.3%)	281 (2.8%)	<0.01
Atrial Fibrillation (N, %)		101 (2.00%)	159 (1.57%)	0.056
CHADS Score (N, %)				<0.01
	0	2915 (57.8%)	7784 (76.9%)	
	1	1213 (24.1%)	1354 (13.4%)	
	2	509 (10.1%)	601 (5.9%)	
	3	226 (4.5%)	220 (2.2%)	
	4	125 (2.5%)	100 (1.0%)	
	5	47 (0.9%)	54 (0.5%)	
	6	6 (0.1%)	8 (0.1%)	
CHADS Score <2 (N, %)		4128 (81.9%)	9138 (90.3%)	<0.01

Note: Continuous variables compared with t-test or nonparametric equivalent; Categorical variables compared with chi-square or nonparametric equivalent; Total cholesterol and HDL cholesterol calculated as peak and nadir, respectively, given several measurements over time

**Table 2:** Odds ratios of atrial fibrillation/flutter by HIV status.

Variable	Model 1	Model 2	Model 3
HIV+ Status	1.28 (0.996–1.65)	1.06 (0.81–1.41)	0.74 (0.55–1.004)
Age		1.08 (1.07–1.09)	1.05 (1.04–1.07)
Male sex		1.82 (1.14–2.90)	2.03 (1.26–3.28)
White		1.0 (ref)	1.0 (ref)
Black		1.16 (0.87–1.54)	0.98 (0.72–1.34)
Hispanic		0.77 (0.43–1.38)	0.67 (0.36–1.25)
Body-Mass index			1.02 (1.004–1.05)
Hypertension diagnosis			2.74 (1.92–3.89)
Diabetes diagnosis			1.95 (1.41–2.70)
COPD diagnosis			2.52 (1.72–3.67)

**Fig 1:**
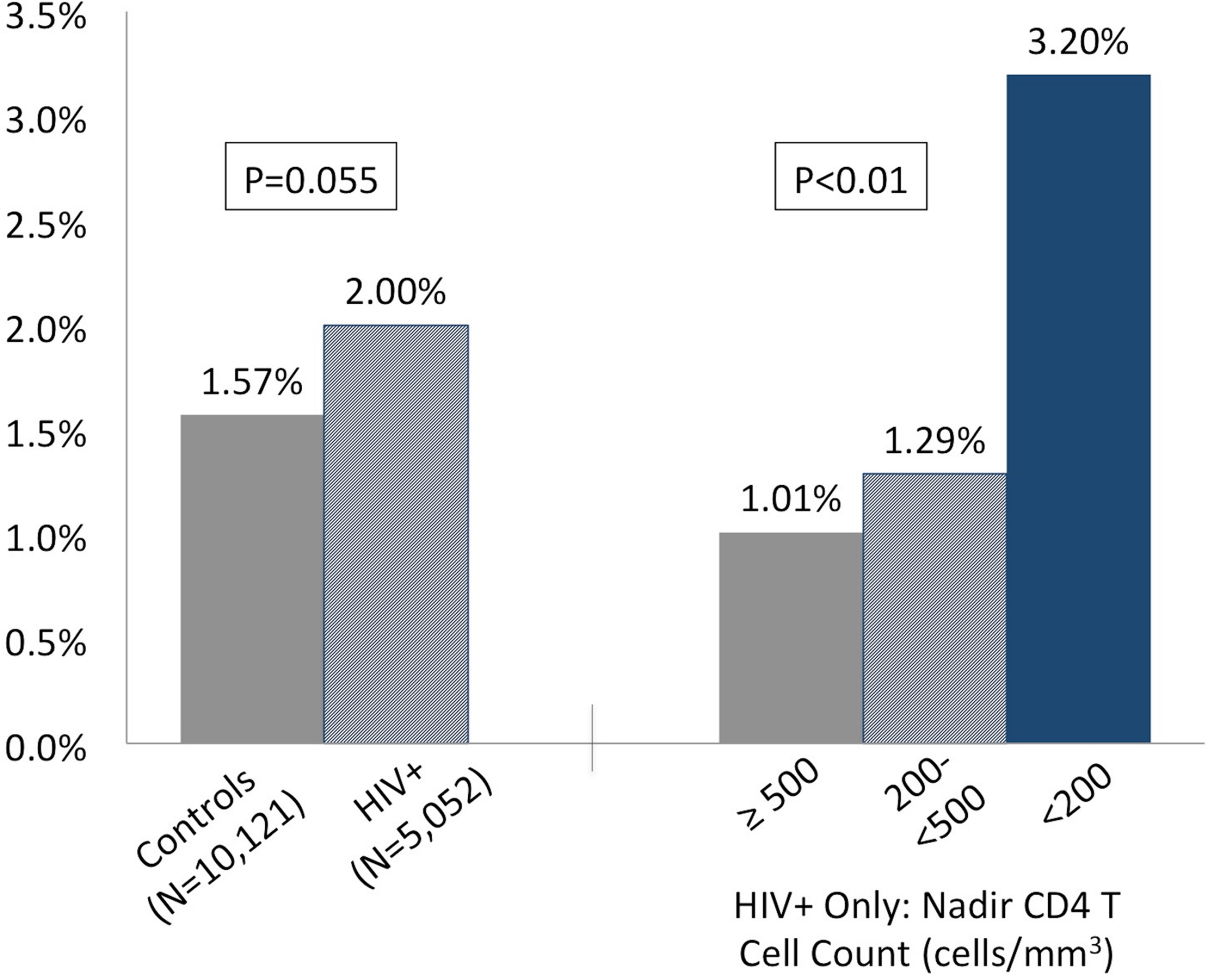
Atrial fibrillation and flutter prevalence for HIV-infected and uninfected persons in HIVE-4CVD.

Among HIV+ persons, nadir CD4 count <200 cells/mm^3^ was associated with approximately two-fold greater odds of AF/AFL; this persisted even after adjustment for age, sex, race, BMI, diabetes, hypertension, smoking, and COPD (Table 3; adjusted OR 1.98, 95% CI 1.21–3.25). Meanwhile, peak HIV viral load was not associated with a significantly elevated odds of AF/AFL (Table 4); a sensitivity analysis comparing HIV+ persons with peak HIV viral load >100,000 copies/mL vs. <500 copies/mL similarly yielded no significant difference in AF/AFL odds (adjusted OR 1.35, 95% CI 0.83–2.20). Interestingly, whereas the proportion of HIV+ persons with CD4+ T cell count <200 with AF/AFL (3.2%) was more than twice that of uninfected controls (1.57%), the proportion of HIV+ persons with higher CD4+ T cell nadir with AF/AFL was actually lower than that of uninfected controls (Fig 1).

**Table 3:** Odds ratios of atrial Arrhythmias for HIV+ persons by CD4 nadir (N = 3355).

Variable	Model 1	Model 2
CD4 nadir <200 cells/mm^3^ (vs. ≥200 cells/mm)	1.99 (1.25–3.17)	1.98 (1.21–3.25)
Age	1.07 (1.05–1.09)	1.04 (1.02–1.07)
Male sex	1.57 (0.77–3.21)	1.58 (0.75–3.30)
White	1.0 (ref)	1.0 (ref)
Black	1.11 (0.69–1.80)	0.89 (0.54–1.47)
Hispanic	0.86 (0.38–1.97)	0.66 (0.27–1.62)
Body-Mass Index (kg/m^2^)		1.00 (0.96–1.05)
Diabetes diagnosis		2.22 (1.31–3.75)
Hypertension diagnosis		2.92 (1.65–5.17)
COPD diagnosis		2.03 (1.13–3.65)

**Table 4:** Odds ratios of atrial Arrhythmias for HIV+ persons by peak HIV VL (N = 2913).

Variable	Model 1	Model 2
Log_10_ of Peak HIV VL (copies/mL)	1.04 (0.86–1.24)	1.03 (0.86–1.24)
Age	1.08 (1.05–1.10)	1.05 (1.02–1.07)
Male sex	1.23 (0.59–2.56)	1.30 (0.61–2.78)
White	1.0 (ref)	1.0 (ref)
Black	1.17 (0.69–1.98)	0.91 (0.53–1.59)
Hispanic	0.79 (0.30–2.06)	0.52 (0.18–1.54)
Body-Mass Index (kg/m^2^)		1.00 (0.95–1.04)
Diabetes diagnosis		2.51 (1.41–4.45)
Hypertension diagnosis		2.76 (1.50–5.08)
COPD diagnosis		2.02 (1.06–3.85)

In addition to low nadir CD4 count, older age, diabetes, hypertension, and COPD were all associated with elevated odds of AF/AFL among HIV+ persons (Tables 3 and 4). Traditional CVD risk factors (age, diabetes, hypertension, and COPD) were also associated with AF/AFL for uninfected controls (S1 Table). Interestingly, whereas elevated BMI was associated with significantly elevated odds of AF/AFL for uninfected controls (S1 Table; OR 1.04, 95% CI 1.01–1.06 for every 1 kg/m^2^ increment in BMI), there was essentially no association between BMI and AF/AFL for HIV+ persons (Tables 3 and 4). In sensitivity analyses in which ART duration was added to multivariable models, each additional year of ART was associated with a slightly lower odds of AF/AFL (S2 Table and S3 Table).

## Discussion

In this study, we found that HIV-associated immune dysfunction (as indicated by nadir CD4+ T cell count <200 cells/mm^3^) was associated with more than twofold greater likelihood of having AF/AFL even after adjustment for demographics and CVD risk factors. We also found that HIV+ patients had elevated odds of AF/AFL when compared to uninfected controls, but that this was attenuated after adjustment for demographic factors and comorbidities and appeared largely driven by HIV+ persons with nadir CD4+ T cell count <200 cells/mm^3^. Old age, hypertension, diabetes, and COPD all were associated with similarly elevated odds of AF/AFL for HIV+ patients and uninfected controls.

We found that HIV-related immune dysfunction (as represented by nadir CD4+ T cell count <200 cells/mm^3^) is associated with more than twofold greater odds of physician-adjudicated AF/AFL among HIV+ persons even after adjustment for demographics and CVD risk factors. Peak HIV viral load, meanwhile, was not associated with AF/AFL among HIV+ persons in this study. Whereas the finding regarding HIV-related immune dysfunction and AF/AFL corroborates a similar finding from the only previous study to our knowledge evaluating AF/AFL among HIV+ persons (conducted in the VA HIV Clinical Case Registry [14], our null finding regarding the HIV viral load and AF/AFL is not in agreement with the VA study.

Our study benefited from the inclusion of an uninfected control group, having a substantial minority of female participants (>17% compared with <3% in the VA study), and using adjudicated AF/AFL endpoints. Thus, it is possible that our different findings regarding HIV viral load and AF/AFL relate to cohort effects or differences in outcome ascertainment. Nevertheless, given the consistent and strong association we found between low nadir CD4+ T cell count and AF/AFL, it may be that immune dysregulation and dysfunction, rather than viremia itself, contributes most to AF/AFL in HIV+ patients.

Atrial remodeling and fibrosis are well-characterized causes of atrial fibrillation [22], and more recent studies have shown that four general types of remodeling lead to the development of AF: electrical, structural, neural, and calcium-related [23]. Systemic diseases with widespread effects (such as diabetes, hypertension, and COPD) and more localized disease processes (such as CAD and MI) are associated with chronic inflammation, myocardial remodeling, and ultimately AF [24, 25]. In HIV, chronic immune dysregulation (as marked by low CD4+ T cell count, among other factors) can lead to chronic inflammation and subclinical CVD [26–29]. Persons with HIV appear to have higher ambulatory blood pressure than uninfected persons and attenuated physiological blood pressure variation, particularly at lower CD4+ T cell counts [30, 31]. Furthermore, diastolic dysfunction and myocardial fibrosis are substantially more common and extensive among HIV-infected persons and may be associated with blood pressure dysregulation, chronic inflammation, immune dysregulation, and/or maladaptive reaction to vascular injury [32–34]. However, given the relative paucity of CVD event data from well-phenotyped HIV cohorts with available biospecimens, precise mechanisms by which HIV-related immune dysfunction and inflammation may lead to CVD remain uncertain. In light of our findings, it is plausible that HIV-related immune dysfunction (as reflected in low nadir CD4+ T cell count) and inflammation increase susceptibility to AF/AFL, but longitudinal studies incorporating biomarker data would be instrumental in further elucidating these mechanisms. Furthermore, studies with adequate power to differentiate between AF and AFL, as well as subtypes of AFL (e.g., right atrial versus left atrial origin) would be useful.

In this study, we also found that AF/AFL was somewhat more prevalent among HIV+ persons compared with frequency-matched controls, but that this difference was attenuated to statistical non-significance after adjustment for demographics and CVD risk factors. This may be due in part to a greater burden of co-morbidity among HIV+ persons compared with uninfected controls, as reflected in the generally sicker nature of the HIV+ persons in our cohort compared with uninfected controls frequency-matched on demographics and zip code. Nevertheless, our finding that greater progression of HIV was associated with a substantially greater likelihood of AF/AFL among HIV+ persons suggests that HIV disease-related factors may also play a role in AF/AFL.

### Strengths and limitations

In our relatively large cohort of HIV+ persons and uninfected matched controls in clinical care, we had the opportunity to review comprehensive clinical data and adjudicate AF/AFL diagnoses. This is the first study to our knowledge to (1) evaluate AF/AFL among HIV+ persons and matched uninfected controls, and (2) analyze adjudicated AF/AFL for HIV+ persons (rather than an administrative code-based endpoint of AF/AFL). These types of analyses are particularly timely given the increasing size and age of the HIV+ population as well as its increasingly apparent elevated risks for various CVD manifestations. Thus, these observational data in a real-life clinical cohort represent a necessary early step to ultimately improving CVD diagnosis, treatment, and prevention among HIV+ persons.

There are several limitations that must be acknowledged. First, as with any electronic health record cohort analysis we are limited by the quality of the data available in the EHR. Although our initial screen included the gold standard ICD-9 and ICD-10 codes, in addition to CPT and word search terms, individual physician documentation is variable, and it is likely that some patients with inadequate documentation of AF/AFL events (particularly out-of-network events) were excluded. However, these limitations were largely unavoidable given the nature of our EHR cohort analyses, and we attempted to account for some of the misclassification through physician adjudication of AF/AFL cases. Second, these analyses were cross-sectional and observational and thus we could not draw causal inferences from our conclusions; we were unable to evaluate time-updated variables or reliable incidence rates of AF/AFL given the structure of our limited electronic dataset. Third, smoking was not included as a covariate in multivariable modeling due to inconsistent availability of these data in our cohort. Given the mildly elevated risk for AF among smokers [35], it is possible that additional adjustment for smoking would have further lowered the multivariable-adjusted odds ratio AF/AFL for HIV+ persons compared with uninfected controls. We were also unable to evaluate alcohol, a known risk factor for AF, as a clinical covariate because these data were not available in our cohort and could not be feasibly collected due to the retrospective nature of this study. Finally, the observation period of 16 years in our study could affect the strength of correlation between HIV status and AF/AFL, as patients may have not received comparable treatments for both HIV and other comorbid conditions.

## Conclusion

We found in a cohort of HIV+ persons and matched, uninfected controls that greater HIV-related immunosuppression is associated with substantially greater odds of AF/AFL among HIV+ persons. Although HIV+ persons had a greater prevalence of AF/AFL than uninfected controls in unadjusted analyses, this was attenuated to non-significance by adjustment for demographics and CVD risk factors and was driven largely by HIV+ persons with nadir CD4+ T cell count <200; HIV+ persons with higher nadir CD4+ T cell counts were no more likely than uninfected controls to have AF/AFL. These findings suggest a potential role of advanced HIV infection and related immunosuppression as a factor associated with AF/AFL for HIV+ patients. Future longitudinal analyses in cohorts with available biorepositories should evaluate potential mechanisms by which HIV-related immune dysfunction may be associated with myocardial fibrosis, dysfunction, and related CVD outcomes including but not limited to atrial arrhythmias.

## Supporting information

S1 Table Odds ratios of demographic and clinical covariates with AF/AFL for uninfected controls. (DOCX)

S2 Table Odds ratios of atrial arrhythmias for HIV+ persons by CD4 nadir and antiretroviral therapy duration. (DOCX)

S3 Table Odds ratios of atrial arrhythmias for HIV+ persons by peak HIV viral load and antiretroviral therapy duration. (DOCX)
