# Supplementary material for: Atrial arrhythmia prevalence and characteristics for human immunodeficiency virus-infected persons and matched uninfected controls
Source: PLoS One. 2018 Mar 20;13(3):e0194754. doi: 10.1371/journal.pone.0194754 (PMC5860783; doi:10.1371/journal.pone.0194754)
Supplement: S1 Table — (DOCX) [file pone.0194754.s001.docx]

S1 Table. Odds Ratios of demographic and clinical covariates with AF/AFL for uninfected controls

| Variable | Odds Ratio for AF/AFL |
| --- | --- |
| Age | 1.06 (1.04-1.08) |
| Male sex | 2.37 (1.26-4.47) |
| White | 1.0 (ref) |
| Black | 1.00 (0.67-1.48) |
| Hispanic | 0.65 (0.27-1.55) |
| Body-mass index (kg/m^2^) | 1.04 (1.01-1.06) |
| Diabetes diagnosis | 1.71 (1.12-2.59) |
| Hypertension diagnosis | 2.66 (1.69-4.18) |
| COPD diagnosis | 2.55 (1.53-4.24 |
