# Supplementary material for: Atrial arrhythmia prevalence and characteristics for human immunodeficiency virus-infected persons and matched uninfected controls
Source: PLoS One. 2018 Mar 20;13(3):e0194754. doi: 10.1371/journal.pone.0194754 (PMC5860783; doi:10.1371/journal.pone.0194754)
Supplement: S2 Table — (DOCX) [file pone.0194754.s002.docx]

S2 Table. Odds Ratios of Atrial Arrhythmias for HIV+ Persons by CD4 Nadir and Antiretroviral Therapy Duration

| Variable |  |
| --- | --- |
| CD4 nadir <200 cells/mm^3^ (vs. ≥200 cells/mm) | 1.93 (1.17-3.18) |
| Age | 1.05 (1.02-1.07) |
| Male sex | 1.72 (0.78-3.79) |
| Black Race (versus white) | 0.83 (0.50-1.40) |
| Hispanic Ethnicity (versus non-Hispanic) | 0.67 (0.27-1.66) |
| Body-Mass Index (kg/m^2^) | 1.01 (0.97-1.06) |
| Diabetes diagnosis | 2.25 (1.32-3.85) |
| Hypertension diagnosis | 2.93 (1.63-5.27) |
| COPD diagnosis | 1.92 (1.05-3.53) |
| ART Duration (years) | 0.91 (0.85-0.97) |
