# Supplementary material for: Atrial arrhythmia prevalence and characteristics for human immunodeficiency virus-infected persons and matched uninfected controls
Source: PLoS One. 2018 Mar 20;13(3):e0194754. doi: 10.1371/journal.pone.0194754 (PMC5860783; doi:10.1371/journal.pone.0194754)
Supplement: S3 Table — (DOCX) [file pone.0194754.s003.docx]

S3 Table. Odds Ratios of Atrial Arrhythmias for HIV+ Persons by Peak HIV Viral Load and Antiretroviral Therapy Duration

| Variable |  |
| --- | --- |
| Log_10_ of Peak HIV VL (copies/mL) | 1.03 (0.86-1.24) |
| Age | 1.06 (1.03-1.08) |
| Male sex | 1.43 (0.64-3.21) |
| Black Race (versus white) | 0.95 (0.54-1.66) |
| Hispanic Ethnicity (versus non-Hispanic) | 0.59 (0.20-1.74) |
| Body-Mass Index (kg/m^2^) | 1.01 (0.96-1.06) |
| Diabetes diagnosis | 2.37 (1.32-4.25) |
| Hypertension diagnosis | 2.73 (1.46-5.10) |
| COPD diagnosis | 1.67 (0.84-3.31) |
| ART Duration (years) | 0.91 (0.85-0.98) |
